# Taurine as a biomarker for aging: A new avenue for translational research

**DOI:** 10.1016/j.abst.2023.10.002

**Published:** 2023

**Authors:** Animesh Acharjee

**Affiliations:** a College of Medical and Dental Sciences, Institute of Cancer and Genomic Sciences, University of Birmingham, B15 2TT, Birmingham, UK; b Institute of Translational Medicine, University Hospitals Birmingham NHS Foundation Trust, B15 2TH, Birmingham, UK; c MRC Health Data Research UK (HDR), Midlands Site, Birmingham, UK; d Centre for Health Data Research, University of Birmingham, B15 2TT, UK

**Keywords:** Biomarker, Diagnostics, Translational research

## Abstract

The physiologic and irreversible process of ageing is accompanied by a wide range of structural and functional shifts at multiple different levels. It is also suggested that variations in the blood concentrations of metabolites, hormones, and micronutrients may play a role in the ageing process. Recently, Singh et al. ^1,2^ investigated a study on Taurine shortage as a driver and biomarker of ageing and its impact on a healthy lifespan.^2^ They further proposed that functional abnormalities in numerous organs associated with age-related illnesses have been linked to early-life Taurine insufficiency. Taurine deficiency in the elderly and the possible benefits of Taurine supplements One of the reasons for decreasing Taurine concentration is the loss of endogenous synthesis, which may contribute to the decrease in Taurine levels seen in the elderly. While it was previously believed that the liver was responsible for most Taurine synthesis in humans, new research suggests that other organs or common intermediates may play a larger role. The authors experimented with and analysed a life-span examination of various organisms, for example, mice to assess the impacts of Taurine supplementation. They also analysed after the administration of oral Taurine supplementation in conjunction with other interventions using multi-omics data sets (RNA sequencing, metabolomics etc.) across different species.

The multifaceted functions of Taurine^1^, ^2^ were thoroughly investigated and also visually explained in Fig. 1, that includes the following major aspects.

1. Taurine may possess anti-inflammatory^3^ properties and facilitate the production of antioxidant enzymes. Additionally, it has the potential to elevate concentrations of antioxidant precursor molecules such as hypotaurine and cysteine.
2. The authors used Taurine supplementation in mice and observed enhanced tolerance to oxidative stress and reduced instances of oxidative damage to DNA.
3. Taurine's further found to be involved in the mitochondrial transfer RNAs. Mitochondrial transfer RNAs also play a significant role in the translation process. And thus, supplementing with Taurine can reverse some of the age-related loss of Taurine in mitochondrial transfer RNAs.
4. Reduced depression-like behaviour and anxiety, Improved glucose homeostasis, improved health span
5. Suppression of senescence suppresses the adverse consequences of telomerase deficiency and DNA damage.
6. Taurine modulates nutrient sensing and proteostasis pathways and improves mitochondrial health.^4^
7. Taurine supplementation improves health parameters in middle-aged nonhuman primates.

**Fig. 1:**
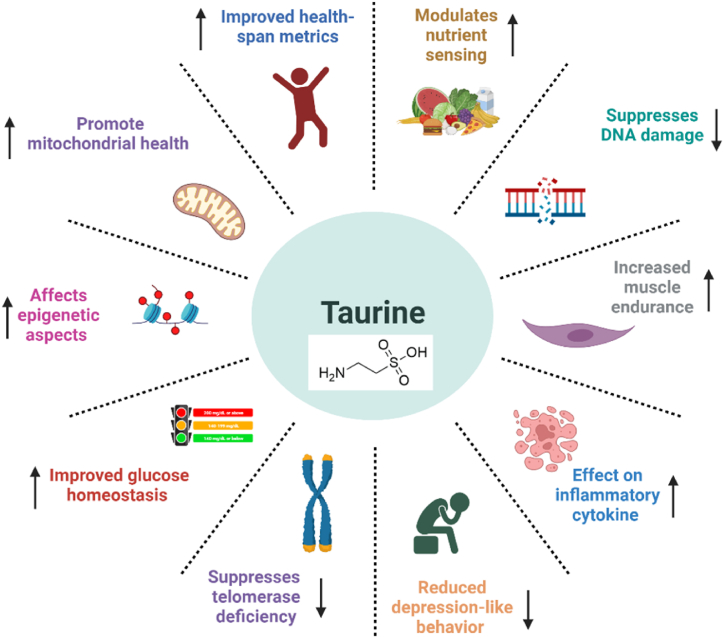
Multiple different functions of the Taurine effecting different biological processes.

In the literature, there are some preclinical studies where Turine was found to be important. for example, Garcia et al., experimented on the Gif gene for absorption, and it was taken out of mice to make a genetic model of B12 insufficiency.^5^^,^^6^ It was found that B12 from the mother is important for growing and controlling bone mass. Administering B12-deficient offspring daily doses of Taurine stopped growth problems and osteoporosis^7^

The biology behind taurine's connection to ageing is very complex and multifactorial. Many processes are involved; for example, chronic inflammation, increased levels of oxidative stress, and mitochondrial dysfunctions are very few of them.^6^ Even though more studies need to be conducted, we are getting closer to understanding the role that Taurine plays and how it influences neurotransmission, growth and sex hormones, bone health, and the composition of the bacteria in the gut. However, targeting Taurine as a biomarker might be an effective therapeutic molecule since it serves multiple functions as well as a diagnostic purpose. It certainly opens a new avenue for translational possibilities^8^ for age-related diseases.

## Funding

The author acknowledge support from the 10.13039/501100000272NIHR Birmingham 10.13039/501100013629SRMRC, HYPERMARKER (Grant agreement ID 101095480), and the 10.13039/501100000265MRC Heath Data Research UK (HDRUK/CFC/01), an initiative funded by 10.13039/100014013UK Research and Innovation, Department of Health and Social Care (England) and the devolved administrations, and leading medical research charities. The views expressed in this publication are those of the authors and not necessarily those of the NHS, the National Institute for Health Research, the Medical Research Council or the Department of Health.

## CRediT authorship contribution statement

**Animesh Acharjee:** Conceptualization, Data curation, Formal analysis, Funding acquisition, Investigation, Methodology, Project administration, Visualization, Writing – original draft, Writing – review & editing.

## Declaration of competing interest

The author declare that he does not have any competing interests.
